# Loneliness during the Pregnancy-Seeking Process: Exploring the Role of Medically Assisted Reproduction

**DOI:** 10.1177/00221465231167847

**Published:** 2023-05-05

**Authors:** Selin Köksal, Alice Goisis

**Affiliations:** 1 University of Essex, Colchester, UK; 2 University College London, London, UK

**Keywords:** Generations and Gender Survey, infertility, loneliness, medically assisted reproduction

## Abstract

This study explores whether undergoing medically assisted reproduction (MAR) is associated with experiencing loneliness and whether this association varies by gender and having a live birth. Using two waves of the Generations and Gender Survey (n = 2,725) from countries in Central and Eastern Europe, we estimate the changes in levels of emotional and social loneliness among pregnancy seekers in heterosexual relationships and test if they vary by the mode of conception while controlling for individual sociodemographic characteristics. Individuals who underwent MAR experienced increased levels of social loneliness compared to individuals who were trying to conceive spontaneously. This association is entirely driven by respondents who did not have a live birth between the two observation periods, while the results did not differ by gender. No differences emerged in emotional loneliness. Our findings suggest that increased social loneliness during the MAR process might be attributable to infertility-related stress and stigma.

Parenthood at later ages is becoming more common, as the mean age at first birth and the share of women giving birth at advanced ages have been steadily increasing since the mid-1980s (Beaujouan 2020; Billari et al. 2007). With parenthood being postponed, many women face longer than expected waiting times before becoming pregnant, an increased risk of subfertility, and an increased need to turn to medically assisted reproduction (MAR)—which include fertility treatments such as ovulation induction, artificial insemination, in-vitro fertilization (IVF), and intracytoplasmic sperm injection (ICSI)—to have a child (Beaujouan et al. 2019; Schmidt et al. 2012).^1^ Access to and availability of MAR have increased markedly over time. Since the first IVF birth in 1978, more than 8 million babies have been born via medical intervention. By 2050, the cumulative number of births via MAR conception is projected to be over 25 million (Faddy, Gosden, and Gosden 2018). As of 2017, 3.1% of the total live births in Europe were conceived through MAR (Wyns et al. 2021).

The growth in the number of individuals undergoing and conceiving through MAR has stimulated research on the consequences of MAR, including on the mental health of those who undergo the treatments. Prior studies have shown that undergoing MAR treatments is an emotionally and physically draining process that can have detrimental effects on women’s mental health because it is associated with increased levels of stress, anxiety, and depression (Hjelmstedt et al. 2003; Huang et al. 2019; Joelsson et al. 2017).

Among the potential drivers of the worsening mental health of couples undergoing MAR is that MAR couples tend to dedicate less time to social relationships and to the quality of their partnership (Nicoloro-SantaBarbara et al. 2018; Tosi and Goisis 2021; Wischmann et al. 2009). This could suggest that feelings of loneliness, broadly defined as perceived deficiencies in both the quality and the quantity of social relationships (De Jong Gierveld, Van Tilburg, and Dykstra 2006), could be linked to undergoing MAR. Individuals who try to conceive via MAR are often exposed to social pressure and infertility stigma (Passet-Wittig and Bujard 2021), which could isolate them from their family, friends, and larger social networks (Peterson et al. 2006). Furthermore, by restricting the time and the financial resources individuals have available to dedicate to friends, family, and leisure activities (Wang et al. 2007), undergoing MAR can increase the risk of feeling lonely. Thus, people who are undergoing MAR treatments may experience reduced partnership quality and satisfaction (Nicoloro-SantaBarbara et al. 2018) and worsening mental health, which are strongly associated with frequent feelings of loneliness (Kearns et al. 2015).

To the best of our knowledge, no prior study has investigated the relationship between undergoing MAR and experiencing loneliness, which is an important gap in knowledge for two main reasons. First, loneliness is a significant aspect of population health and an indicator of social well-being that can exacerbate the risk of morbidity and mortality (Fekete, Williams, and Skinta 2018). The vast majority of the loneliness literature focuses on individuals in mid and later life, given the higher prevalence of loneliness in that period of the life course. However, loneliness can also be experienced earlier in life (Beutel et al. 2017; Nyqvist et al. 2016). Identifying critical stages of the life course, such as when seeking to conceive and undergoing MAR, when individuals might be at higher risk of experiencing loneliness is important, especially given that feeling lonely earlier in life can translate into poorer physical and mental health outcomes later in life.

Therefore, exploring the association between MAR and loneliness enriches our understanding of the early life pathways into loneliness and the effects of experiencing infertility and undergoing MAR. Moreover, the findings on loneliness can provide further insights on how MAR is associated with other health conditions, such as mental health, because the social and the emotional support that social relationships provide are central to and protective of mental health (Turner and Brown 2010). Loneliness, as a precursor of poorer mental well-being, could be integral to the association between undergoing MAR and mental health.

Second, investigating the link between loneliness and MAR can contribute to our understanding of infertility as a socially constructed state as opposed to an objectively defined one. Infertility represents not only a medical diagnosis but also a lack of a desired status or a social role. Undergoing MAR requires individuals to negotiate not only with their partners but also with their families and larger social networks during and even after the treatments (Greil, Slauson-Blevins, and McQuillan 2010). These within-couple and external negotiations take place in contexts that are shaped by reproductive norms. Deviations from those norms can result in informal sanctions in the form of alienation and lack of social support that can be harmful in terms of well-being (Huijts, Kraaykamp, and Subramanian 2013). Looking at loneliness, unlike other well-being indicators (e.g., mental health, subjective well-being etc.), enables us to decompose and compare how the processes of infertility and MAR are shaped by intimate relationships and the wider social context because it can be experienced and measured both in terms of lack of close emotional contacts and a wider social network of support.

Overall, studying whether and, if so, how MAR is associated with loneliness contributes to the field of medical sociology not only by enhancing our understanding of the social construction of health and illness and its well-being implications but also by enriching our understanding of the ways in which social ties and social norms shape the medical treatment experience. Moreover, by documenting whether the treatment outcome is integral to one’s social status and connectedness, this study is well placed to inform and enrich theories and research on the psychosocial consequences of medical treatments.

Using data on Central and Eastern European countries from the Generations and Gender Survey (GGS),^2^ we make a twofold contribution to the literature. First, we explore the relationship between undergoing MAR and loneliness by investigating whether the pregnancy-seeking process is differentially associated with changes in individuals’ feelings of emotional and social loneliness by the mode of conception (MAR vs. spontaneous conception). We also explore whether this relationship varies by gender and by whether the pregnancy-seeking process did or did not result in a live birth. While the social science literature on the role of men in reproduction has been growing, a large share of previous studies on infertility and MAR treatments focused on the female experience, in part because MAR medical knowledge and practices have centered on female infertility (Halcomb 2018). Moreover, little is known about how undergoing fertility treatments as a heterosexual couple affects men’s well-being even though couple dynamics and relationship quality are among the crucial determinants of loneliness. This lack of research on men’s experiences with MAR not only leads to knowledge gaps, but it also reinforces gender inequality in reproductive responsibilities by suggesting that men play a secondary role in reproductive matters. At the same time, only a few existing studies have explored whether the pregnancy-seeking process results in a live birth or how this process moderates the outcome of interest even though the success rates of fertility treatments are relatively low (ESHRE Task Force on Ethics and Law et al. 2010). The outcome of the treatment process is a relevant aspect to consider given that the social expectations regarding childbearing and the stigma surrounding infertility can put couples under pressure and may increase their risk of feeling lonely.

The second contribution of this article is methodological. Previous studies on MAR and childlessness have relied on cross-sectional data sources, which are prone to simultaneity bias (Greil et al. 2019). To address this limitation, we draw on the two-wave longitudinal data of the GGS, which allow us to rule out potential time-invariant confounding factors that are correlated with both undergoing MAR and experiencing loneliness. Moreover, by making use of panel data, we can address potential selection issues related to differences in the socioeconomic backgrounds and the baseline loneliness levels of individuals in heterosexual couples who are seeking to conceive through MAR and those who are trying to conceive spontaneously. While couples who undergo MAR have, on average, higher levels of education and income (Barbuscia, Myrskylä, and Goisis 2019), MAR patients might be lonelier at the baseline because they are feeling distress and stigma due to their infertility. We mitigate these empirical concerns by running a multivariate analysis that controls for individuals’ baseline levels of loneliness and sociodemographic characteristics.

## Background

### MAR and Loneliness

According to De Jong Gierveld et al. (2006), loneliness is an unpleasant or inadmissible feeling that arises when a person lacks a sufficient quantity or quality of interpersonal relationships. Thus, loneliness might result from either the number of social relationships an individual has being smaller than the person wants or from a lack of intimacy in the individual’s existing relationships. There are two main types of loneliness: *emotional loneliness*, stemming from the absence of close emotional attachments (e.g., a partner or a best friend), and *social loneliness*, stemming from the lack of a broader group of contacts or engagement in a larger social network (e.g., family, friends, colleagues, neighbors; Weiss 1973). Feelings of emotional loneliness tend to intensify when a person experiences a deterioration in relationship quality or the dissolution of a union, while feelings of social loneliness tend to arise when an individual lacks wider social networks in which the person feels accepted and welcomed. Loneliness can also arise as a consequence of individuals’ responses to their social situation, which may be influenced by their recent life events and the availability of opportunities (Kearns et al. 2015).

**Figure 1.**
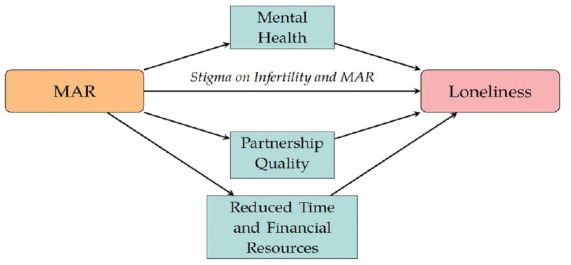
Summary of the Mechanisms. *Note*: MAR = medically assisted reproduction.

Figure 1 illustrates the mechanisms through which undergoing MAR could be linked to feelings of loneliness. Undergoing MAR could be *directly* associated with loneliness because the experience of infertility may be related to a strong sense of stigmatization and social isolation due to the person’s inability to fulfill social expectations regarding parenthood (Greil et al. 2010; Johansson and Berg 2005). Because loneliness has been linked to a lack of acceptance by the community in which an individual lives (Beutel et al. 2017), couples who are undergoing MAR might be at higher risk of experiencing loneliness and social isolation. Moreover, because individuals who feel stigmatized by their environment can find building and maintaining social relationships challenging (Hsieh and Liu 2021), the stigmatization of infertility could lead MAR patients to avoid engaging in self-disclosure or seeking social support (Faccio, Iudici, and Cipolletta 2019; Passet-Wittig and Bujard 2021).

Undergoing MAR could be *indirectly* related to loneliness via three mechanisms. First, it may be related through relationship quality because couples who are undergoing MAR are more likely than couples who are trying to conceive spontaneously to experience relationship instability or a reduction in the quality of their relationship due to the prolonged nature and the demands of the treatment process (Holter et al. 2006; Wang et al. 2007). Second, MAR treatments are costly in terms of time and money, and couples might find it challenging to reconcile their professional lives with frequent doctor visits and the strict requirements of MAR treatment protocols (Courbiere et al. 2020). Therefore, undergoing MAR can reduce the resources individuals have available to dedicate to friends, family, and leisure activities (Nicoloro-SantaBarbara et al. 2018; Parry and Shinew 2004; Tosi and Goisis 2021). Third, a person’s mental health status could mediate the association between undergoing MAR and feeling lonely. Previous studies have shown that individuals who are undergoing MAR often have increased feelings of anxiety and stress due to the adverse effects of hormonal therapies and the relatively low success rates of the treatments (Hjelmstedt et al. 2003; Huang et al. 2019; Verhaak et al. 2007) or because they have a bodily deficiency that is medically diagnosed and legitimated (Greil 1997; Jacob, McQuillan, and Greil 2007; Johnson and Fledderjohann 2012; Jutel 2009). Furthermore, poorer mental health and chronic stress, anxiety, and depression are strongly associated with frequent feelings of loneliness (Kearns et al. 2015). Hence, we expect individuals who are trying to conceive via MAR will experience a stronger increase in loneliness than individuals who are trying to conceive spontaneously (Hypothesis 1a).

On the other hand, MAR patients represent a selected subpopulation (Inhorn and Birenbaum-Carmeli 2008) because they tend to have higher levels of education and income than individuals who conceive spontaneously. This could be because individuals with higher socioeconomic status (SES) are more likely to postpone childbearing and are thus more likely to need medical treatments to overcome infertility, which increases with age (Räisänen et al. 2013). Moreover, in many contexts, MAR treatments can be costly and are thus more easily accessed by higher-income groups (Präg and Mills 2017). In terms of coping with loneliness, individuals with higher SES have more resources and better access to mental health and medical support, which could help them reduce their risk of feeling lonely (Allen et al. 2014). Compared to their less advantaged counterparts, individuals with higher SES tend to have more social capital, higher levels of social engagement, wider social networks, and higher levels of trust in the society they live in, which can protect them from feeling socially lonely (Nyqvist et al. 2016). Alternatively, we do not expect individuals’ feelings of loneliness to differ depending on whether they are trying to conceive via MAR or are trying to conceive spontaneously (Hypothesis 1b).

Next, we discuss how gender and the outcome of the pregnancy-seeking process (i.e., whether it ends in a live birth) can modify the association between undergoing MAR and experiencing loneliness through the direct and the indirect mechanisms outlined in Figure 1.

### Variation by Gender

Because reproduction is a gendered experience that mainly occurs in women’s bodies, fertility treatments are disproportionately designed to overcome female infertility (Almeling 2015; Inhorn and Birenbaum-Carmeli 2008). The gendered context of reproduction also manifests itself in differences in the social expectations regarding parenthood for women and for men. As pronatalism remains a dominant ideology in today’s societies, parenthood is perceived as more socially binding for women than for men, and motherhood is perceived as an almost indispensable component of female identity (Bell 2019). Furthermore, the availability of MAR per se, and thus the possibility of overcoming infertility, might even reinforce the “motherhood mandate” (Inhorn and Birenbaum-Carmeli 2008; Thompson 2002).

Evidence from empirical studies has validated the gendered expectations of childbearing and the gendered implications of undergoing MAR. For instance, women bear a larger physical burden than men when undergoing MAR, and they have stronger psychological reactions during the treatments (Hjelmstedt et al. 1999). Because gendered socialization regarding procreation affects the way infertility is perceived, women who are undergoing MAR tend to report higher levels of infertility-related stigma than their male partners (Slade et al. 2007), which could, ultimately, lead them to feel more socially lonely and isolated. Therefore, we hypothesize that women who undergo MAR experience a stronger increase in loneliness than their male counterparts to (Hypothesis 2a).

On the other hand, because undergoing MAR can be a challenge for both partners in a couple, the man and the woman could be similarly influenced by the process. It has been shown that partners who undergo MAR are equally emotionally stressed while sharing similar hopes and positive feelings for the future (Boivin et al. 1998). If a couple fails to achieve a pregnancy at the end of MAR treatments, the male partner is roughly as likely as the female partner to experience a short-term decline in emotional well-being (Holter et al. 2006). Tosi and Goisis (2021) found that the mental and the subjective well-being of both partners in a couple tend to worsen while they are undergoing MAR treatments and tend to recover when they become parents via MAR. It has also been suggested that partnership quality is an important mechanism in a couple’s mutual experience of undergoing MAR that could render both partners emotionally lonely, which might lead to relationship dissatisfaction or dissolution. Hence, alternatively, we expect that women and men who undergo MAR experience a similar increase in loneliness (Hypothesis 2b).

### Variation by Live Birth

Although MAR is considered an innovative and effective solution for individuals who experience infertility, its success rate (i.e., the share of cycles that result in a live birth) remains relatively low (ESHRE Task Force on Ethics and Law et al. 2010). For example, a population-based study conducted in the UK found that the share of women who had a live birth after undergoing IVF treatment, one of the available MAR techniques, was 31.2% after the first cycle and 57.1% after three cycles (McLernon et al. 2016). Due to the low success rates of MAR treatments and given the significant likelihood of enduring repeated cycles of unsuccessful attempts, most individuals who undergo MAR experience an “emotional rollercoaster” and “never enough” feelings (Greil et al. 2010). Therefore, when exploring individuals’ feelings of loneliness during the MAR process, it is essential to take into account the outcomes of the treatments they underwent.

Because parenthood remains central to most people’s social identities, the transition to “non-parenthood” can be a stressful experience (Holter, Bergh, and Gejervall 2021; McQuillan et al. 2003). Women who failed to conceive after undergoing (often several) treatments were more likely to suffer from distress, anxiety, depression, and feelings of being lost and lonely than women whose fertility treatments resulted in a live birth (Holter et al. 2021; Milazzo et al. 2016). Before the partners in a couple define themselves as infertile and permanently childless, they tend to engage in negotiations not only with medical professionals but also within their partnership and their larger social environment (Greil, McQuillan, and Slauson-Blevins 2011). Due to the unexpected stressors and potential stigmatization of infertility, couples may experience changes in their social networks and family relationships and even potential threats to their future together (Peterson et al. 2006). Moreover, given that the desire to have a child is perceived as a norm in most societies, childlessness can be considered a “deviant behavior,” which might prevent couples from seeking social support (Slade et al. 2007). As a consequence, individuals’ feelings of loneliness may be expected to vary depending on whether their MAR treatments do or do not result in a live birth. Thus, we anticipate that individuals whose MAR treatments do not result in a live birth experience stronger increase in loneliness than the individuals whose MAR treatments end in a live birth (Hypothesis 3).

## Data and Methods

### Analytical Sample

We made use of the first two waves of the Generations and Gender Survey (GGS), which were conducted in 2004 to 2011 and in 2007 to 2015, respectively.^3^ The GGS is a longitudinal and nationally representative survey that collects retrospective information on numerous sociodemographic indicators, including on fertility, fecundity, and fertility treatments, from individuals ages 18 to 80 living in European countries (Vikat et al. 2007). The time interval between the two waves was three years for all of the participating countries.

We included in our analysis countries that collected information on loneliness and the mode of conception in both waves: namely, Bulgaria, Georgia, Germany, Austria, and Poland.^4^ We identified respondents who were partnered and were not pregnant and who wanted to have a baby at the time of the interview in Wave 1 (n = 2,822).^5^ Among these respondents, 2,725 (97%) had complete information on mode of conception and loneliness variables. This constituted the final number of observations of the analytical sample. It should be noted here that the GGS’s fertility and fecundity modules were collected from female respondents or from male respondents with female partners who were under age 50 and who had already had sexual intercourse with a person of the opposite sex. In addition, the question regarding the mode of conception was asked of respondents who had a coresident or a nonresident partner at the time of the survey. Due to the way the fertility and fecundity questionnaire is designed in GGS, our sample consists of individuals who are reported to be in heterosexual partnerships. Because the data do not contain any information on respondent’s gender identity, we cannot identify whether the individual is cisgender, transgender, or nonbinary.

Among other variables used in the analyses, we remark that education and subjective financial hardship variables contain 19 (1%) and 37 (1.4%) missing cases (see Table 1). Following Graham, Olchowski, and Gilreath’s (2007) recommendations on the number of imputations, we ran 20 imputations using using the multiple imputation strategy to replace the missing values.

**Table 1.** Descriptive Statistics of the Variables Introduced in the Main Analysis by Mode of Conception (*N* = 2,725).

	SC	MAR	Difference		
	Mean		*p* Value	*N*	Missing (%)
Panel A					
Loneliness scale					
Emotional loneliness (W1)	.46	.54	.23	2,725	0
Emotional loneliness (W2)	.48	.53	.45	2,725	0
Social loneliness (W1)	1.16	1.16	.94	2,725	0
Social loneliness (W2)	1.20	1.39	.07	2,725	0
Overall loneliness (W1)	1.63	1.70	.58	2,725	0
Overall loneliness (W2)	1.68	1.92	.09	2,725	0
Demographics					
Have a child (W1)	.68	.34	.00	2,725	0
Age (W1)	31.7	33.3	.00	2,725	0
Education				2,706	1
Less than secondary education	.08	.08	.93	243	
Secondary education	.57	.55	.59	1,554	
Tertiary education	.35	.37	.61	909	
Mediating factors					
Subjective financial hardship	.32	.38	.16	2,725	0
Union dissolution	.14	.20	.10	2,688	1.4
Interaction variables					
Female	.45	.66	.00	2,725	0
Live birth	.44	.41	.42	2,725	0
Panel B					
Country				2,725	0
Bulgaria	.95	.05	.00	775	
Georgia	.93	.07	.00	809	
Germany	.85	.15	.00	220	
Austria	.93	.07	.00	472	
Poland	.95	.05	.00	449	
*N*	2,527	198			

*Source*: Generations and Gender Survey Wave 1 (2004–2011) and Wave 2 (2007–2015).

*Note*: SC = spontaneous conception; MAR = medically assisted reproduction; *N* = number of observations; W1 = Wave 1; W2 = Wave 2.

### Dependent Variable

The dependent variable was a shortened six-item version of the De Jong Gierveld Loneliness Scale, which provides reliable measurements of overall, emotional, and social loneliness (De Jong Gierveld et al. 2006). The reliability and validity of the overall loneliness scale and emotional and social loneliness subscales were also tested using GGS data (De Jong Gierveld and Van Tilburg 2010). This scale consisted of six items: The first three were designed to measure emotional loneliness, while the remaining three were designed to measure social loneliness. The total score for the six items yielded the overall loneliness score. Emotional loneliness, which stems from the lack of an intimate relationship or a close emotional attachment (De Jong Gierveld and Van Tilburg 2006), was measured through the following statements: (1) “I experience a general sense of emptiness,” (2) “I miss having people around,” and (3) “Often, I feel rejected.” The response options were “yes,” “more or less,” and “no.” For each statement regarding emotional loneliness, positive (yes) and neutral (more or less) answers were assigned 1 point, while negative (no) answers were assigned 0 points. Social loneliness, which arises from the lack of a broader social network (e.g., friends, colleagues, relatives, and neighbors; De Jong Gierveld and Van Tilburg 2006), was quantified via following propositions: (4) “There are plenty of people that I can lean on in case of trouble,” (5) “There are many people that I can count on completely,” and (6) “There are enough people that I feel close to.” While the response options were the same as those for emotional loneliness, negative (no) and neutral (more or less) answers to each of these statements were assigned 1 point, while positive (yes) answers were assigned 0 points. Both the emotional and the social loneliness subscales ranged from 0 (not lonely) to 3 (severely lonely). Finally, the overall loneliness score, which ranged from 0 (not lonely) to 6 (extremely lonely), was the sum of the emotional and the social loneliness subscales. The questions on loneliness were asked both in Waves 1 and 2.

### Independent Variable

The main independent variable identified the mode of conception at Wave 1 through the following question: “Are you (or your current partner/spouse) doing any of the things listed on this card to help you (your partner or spouse) get pregnant?” We generated a MAR dummy that took the value of 1 if at the time of Wave 1 interview the respondent or the partner of the respondent was doing any of the following at time of the interview: (1) receiving medication, (2) IVF or micro-fertilization (ICSI), (3) surgery, (4) artificial insemination, and (5) other medical treatment. The dummy took the value of 0 (i.e., seeking pregnancy spontaneously) if the respondent or the partner of the respondent was not doing any of these things to help them get pregnant.^6^

### Control Variables and Mediating Factors

We controlled for variables that can confound the association between the mode of conception and loneliness. As baseline individual characteristics, we adjusted for a set of sociodemographic factors that included age (and age squared), gender, educational attainment (1 = less than secondary level, 2 = secondary education, 3 = tertiary education), parental status (0 = no child, 1 = at least one child) in Wave 1, and country of residence.

Moreover, we adjusted for two potential mediators (see “Background” section and Figure 1): *subjective financial distress* (as an indicator of reduced time and financial resources) and *union dissolution* (as an indicator of partnership quality). We constructed the subjective financial hardship variable using answers to the question, “Thinking of your household’s total monthly income, is your household able to make ends meet?” The question had the following possible responses: (a) “with great difficulty,” (b) “with difficulty,” (c) “with some difficulty,” (d) “fairly easily,” (e) “easily,” and (f) “very easily.” Based on these responses, we generated a dummy variable that takes the value of 1 if the household’s subjective financial capability worsened between the two waves and takes the value of 0 if the household’s subjective financial capability remained stable or improved between the two waves. The union dissolution dummy variable was coded as 1 if the respondent underwent a union dissolution in between the two waves and as 0 otherwise.

### Interaction Variables

We examined two factors that may lead the relationship between loneliness and the mode of conception to vary. First, we explored whether feelings of loneliness differed by gender. To do so, we introduced a dummy that takes the value of 1 if the respondent was female and the value of 0 if the respondent was male. Second, we tested whether the respondent’s pregnancy-seeking process did or did not result in a least one live birth. To do so, we compared the number of children reported in Wave 1 and in Wave 2 and generated a dummy variable that takes the value of 1 if the respondent had a child between the two waves and the value of 0 otherwise. These two variables were interacted with the MAR dummy and included in the model as interaction terms in separate steps of the analysis.

### Contextual Variables

The respondents in Wave 1 of the GGS were presented with a list of statements designed to measure their opinions of how their social environment has influenced their childbearing. The respondents were asked to what extent they agreed with the following statements: “Most of your friends think that you should have a/another child,” “Your parents think that you should have a/another child,” and “Most of your relatives think that you should have a/another child.” The possible answers ranged from “strongly disagree” (1) to “strongly agree” (5). The subjective nature of the question can shed some light on how the respondents perceived the views on childbearing of their social networks. However, because the question was asked only at the baseline survey, we were not able to track the changes between the two waves in the respondents’ perceptions of the social pressure to have a child or to provide a breakdown by whether the respondents did or did not have a live birth. Nonetheless, to contextualize the MAR experience, we descriptively analyzed the level of perceived social pressure to have a child by the mode of conception at the baseline of the main analyses.

### Empirical Strategy

As a first step, we estimated whether undergoing MAR to conceive was associated with larger changes in emotional, social, and overall loneliness than conceiving spontaneously (Hypotheses 1a and 1b). We used an ordinary least squares estimator with a lagged dependent variable. In Model 0—the baseline model—we estimated the effect of the mode of conception on loneliness at Wave 2 by controlling for the baseline (Wave 1) level of loneliness. In Model 1, we included adjustments for sociodemographic characteristics and country fixed effects. In the following, we present the equation of Model 1:

$$\begin{array}{l} Loneliness_{i2}=\beta_{1}MAR_{i1}+\beta_{2}Loneliness_{i1}\\ \;\;\;\;\;\;\;\;\;\;\;\;\;\;\;\;\;\;+\beta_{3-7}X_{i1}+\gamma_{c}+\varepsilon_{i}, \end{array}$$

where *Loneliness_i2_* stands for the emotional, social, or overall loneliness scale in Wave 2; *MAR_i1_* indicates the dummy for undergoing MAR in Wave 1; *Loneliness_i1_* represents the baseline emotional, social, or overall loneliness scale; *X_i1_* represents sociodemographic controls, such as age, age squared, educational level, gender, and number of children; *γ_c_* is the country fixed effects (country dummy variables) of the country of residence at Wave 1; and *ε_i_* stands for the idiosyncratic error term. In Models 2 to 4, we additionally controlled for the time-varying mediators *subjective financial hardship* and *union dissolution*.

In a second step, we estimated whether the association between the mode of conception and the feeling of loneliness varied by gender (Hypotheses 2a and 2b) or by whether the pregnancy-seeking process did or did not result in a live birth (Hypothesis 3). For the first step, we report the MAR coefficient. For the second step, we report the marginal effect of not having given birth to at least one child and being female compared to having given birth to at least one child and being male.

Attrition in the GGS was relatively high. For our sample, we calculated the attrition rate between Wave 1 and Wave 2 as 37%. To correct for potential bias due to attrition, we weighted our sample with country-specific weights provided in Wave 2 that were standardized by GGS based on the country-specific population weights. These weights allowed us to adjust the sample in terms of age, sex, household structure, and region at baseline (Fokkema et al. 2016) and to correct the estimates for the attrition rates of population subgroups (Tosi and Grundy 2019).

## Results

### Descriptive Results

Table 1 reports the statistics of all variables used in the main analysis by mode of conception. When we look at Panel A, we see that feelings of emotional and social loneliness did not vary by mode of conception at Wave 1. On the other hand, when we look at the loneliness scale at Wave 2, we see that the level of social loneliness of individuals who underwent MAR was slightly higher than that of spontaneous conceivers (*p* = .07). Moreover, it appears that the educational levels of respondents who conceived spontaneously or via MAR did not differ substantially. This finding is not in line with the previous literature, which reported that individuals who undergo MAR tend to be socioeconomically advantaged even in countries where fertility treatments are highly subsidized (Barbuscia et al. 2019; Goisis et al. 2020). However, this evidence is largely based on couples who had a live birth, which could conceal socioeconomic differences between the couples who had an unsuccessful MAR attempt and the couples who conceived via MAR (Köppen, Trappe, and Schmitt 2021). Indeed, the latter may be expected to have higher SES because conceiving through MAR can require several attempts that are costly in terms of money and time. In Appendix Table 2 in the online version of the article, we show that this was indeed the case for our analytical sample: The couples who conceived through MAR had higher educational levels than the couples who underwent MAR but did not have a baby and the couples who were trying to conceive spontaneously.

In terms of mediating factors, the mean experience of subjective financial hardship and union dissolution did not differ statistically by the mode of conception.

Regarding the interaction variables, 41% of the respondents who underwent MAR and 45% of the respondents who were trying to conceive spontaneously had a baby between the two waves. Moreover, 66% of the respondents who underwent MAR were women, while only 45% of the respondents who were trying to conceive spontaneously were female. The gender imbalance in MAR conception is notable, and additional analyses suggest that it can be explained by (a) men being more likely to skip the question on the mode of conception and (b) men being more likely to say that their partner does not use or do anything to help her get pregnant (see Appendix Table 1 in the online version of the article).

In Panel B of Table 1, we present the percentages of respondents who were trying to conceive spontaneously and via MAR by the country of residence. Unsurprisingly, the percentage of respondents who were trying to conceive spontaneously was significantly higher than the percentage of respondents who were trying to conceive via MAR in each country. On the other hand, the percentage of individuals who were trying to conceive via MAR was markedly higher in Germany, which can be attributed to Germany having policies that provide easier access to fertility treatments than the other countries in our sample.

**Figure 2.**
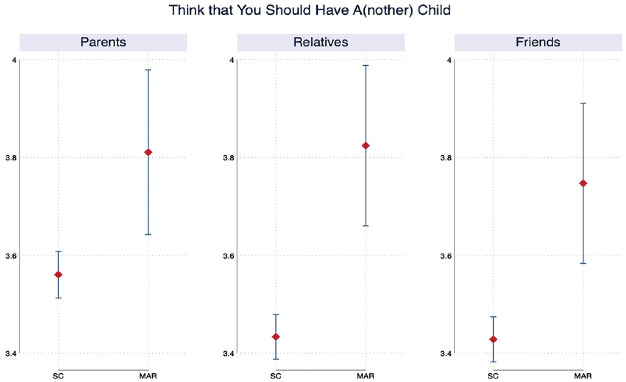
Perceived Social Pressure on Childbearing (with 95% Confidence Interval; *N* = 2,725). *Source*: Generations and Gender Survey Wave 1 (2004–2011) and Wave 2 (2007–2015). *Note*: SC = spontaneous conception; MAR = medically assisted reproduction.

For context, we test whether the perceived social pressure on childbearing varies by the mode of conception. Figure 2 shows that the proportion of respondents who agrees that their parents, relatives, and friends think that they should have a/another child is significantly higher among individuals who were undergoing MAR than it is among individuals who were trying to conceive spontaneously. This may be because individuals who are undergoing MAR are, on average, older (see Table 1; *p* = .00) and had fewer children (see Table 1; *p* = .00) than individuals who were trying to conceive spontaneously. Alternatively, it could be because individuals who were undergoing MAR due to subfertility were often taking a long time to conceive, which prompted their network of families and friends to put pressure on them. Similarly, childlessness could have become a salient issue for individuals who were undergoing MAR because they invested considerable time and financial resources to conceive and they might have become more sensitive to social pressure on childbearing coming from their close social network.

### Changes in Loneliness in between the Two Waves

Table 2 reports the results obtained by estimating Model 0 and Model 1. Model 0, which only adjusts for the relevant baseline loneliness level, shows that undergoing MAR was associated with an increase in social loneliness of almost .19 points (significant at 5% level). The association remains robust (.2-point increase, significant at the 5% level) when we include adjustments for sociodemographic controls in Model 1. For social loneliness, the findings support Hypothesis 1a, which predicts that the individuals who underwent MAR will experience stronger increase in loneliness compared to individuals who tried to conceive spontaneously. By contrast, for emotional and overall loneliness, the results support Hypothesis 1b, which postulates that the feeling of loneliness does not depend on the mode of conception.

**Table 2.** The Association between the Mode of Conception and Changes in Loneliness (*N* = 2,725).

	(M0)	(M1)	(M0)	(M1)	(M0)	(M1)
	Emotional	Emotional	Social	Social	Overall	Overall
MAR (reference = spontaneous conception)	.029 (.062)	–.028 (.063)	.186* (.085)	.196* (.085)	.206^+^ (.117)	.166 (.118)
Education (reference = less than secondary)						
Secondary		–.135* (.062)		–.163* (.082)		–.268* (.115)
Higher		–.278** (.066)		–.211* (.087)		–.446** (.123)
Country (reference = Bulgaria)						
Georgia		.255** (.048)		.102 (.065)		.335** (.090)
Germany		.112^+^ (.064)		–.586** (.087)		–.436** (.121)
Austria		–.114* (.054)		–.728** (.074)		–.792** (.102)
Poland		–.056 (.046)		–.393** (.062)		–.413** (.086)
Children (1 = have a child)		–.045 (.036)		.017 (.048)		–.027 (.067)
Age		.035^+^ (.019)		.059* (.025)		.093** (.035)
Age^2^		–.000 (.000)		–.001^+^ (.000)		–.001* (.001)
Gender (1 = female)		.051 (.032)		–.065 (.043)		–.017 (.060)
Emotional loneliness (W1)	.278** (.019)	.243** (.019)				
Social loneliness (W1)			.362** (.018)	.260** (.019)		
Overall loneliness (W1)					.396** (.018)	.308** (.019)
R^2^	.072	.105	.131	.196	.151	.205
Observations	2,725	2,725	2,725	2,725	2,725	2,725

*Source*: Generations and Gender Survey Wave 1 (2004–2011) and Wave 2 (2007–2015).

*Note*: The coefficients are estimated via ordinary least squres. Standard errors are in parentheses. MAR = medically assisted reproduction; W1 = Wave 1; M0 = Model 0; M1 = Model 1.

+ *p* < .1, *p < .05, **p < .01 (two-tailed tests).

Next, we introduce two mediating factors, *union dissolution* and *subjective financial hardship*, in a stepwise approach (see Table 3). First, in Model 2, we introduce the experience of union dissolution between the two waves and find that changes in social loneliness were not attenuated compared to Model 1 (from .2 to .19 points, significant at the 5% level). In Model 3, we find that while subjective financial hardship was positively and significantly associated with emotional and overall loneliness, it did not explain changes in social loneliness because the coefficient is very similar to the one reported in Model 1. Lastly, after both mediators are simultaneously included in Model 4, the results from previous estimates hold (.19 points, significant at the 5% level for social loneliness).

**Table 3.** The Association between the Mode of Conception and Changes in Loneliness (with Mediating Factors; *N* = 2,725).

	(M2)	(M3)	(M4)	(M2)	(M3)	(M4)	(M2)	(M3)	(M4)
	Emotional	Emotional	Emotional	Social	Social	Social	Overall	Overall	Overall
MAR (reference=spontaneous conception)	–.029 (.063)	–.032 (.063)	–.033 (.063)	.194* (.085)	.194* (.085)	.192* (.085)	.162 (.118)	.160 (.118)	.157 (.118)
Union dissolution	.154** (.047)		.148** (.047)	.211** (.063)		.208** (.063)	.360** (.088)		.350** (.088)
Subjective financial hardship		.092** (.033)	.087** (.033)		.050 (.045)	.043 (.045)		.153* (.062)	.141* (.062)
Education (reference = less than secondary)									
Secondary	–.125* (.062)	–.144* (.062)	–.130* (.062)	–.145^+^ (.082)	–.167* (.082)	–.148^+^ (.082)	–.240* (.115)	–.280* (.115)	–.248* (.115)
Higher	–.270** (.066)	–.286** (.066)	–.274** (.066)	–.196* (.087)	–.214* (.087)	–.198* (.087)	.424** (.122)	.457** (.123)	.431** (.122)
Country (reference = Bulgaria)									
Georgia	.260** (.048)	.243** (.049)	.249** (.048)	.108^+^ (.065)	.096 (.065)	.103 (.065)	.347** (.090)	.317** (.091)	.329** (.091)
Germany	.083 (.065)	.105 (.064)	.076 (.065)	.627** (.088)	.589** (.087)	.630** (.088)	.506** (.122)	.446** (.121)	.515** (.121)
Austria	–.153** (.055)	–.116* (.053)	–.153** (.055)	.781** (.075)	.728** (.074)	.780** (.075)	.883** (.104)	.792** (.102)	.881** (.104)
Poland	–.065 (.046)	–.063 (.046)	–.072 (.046)	.407** (.062)	.397** (.062)	.410** (.062)	.437** (.086)	.424** (.086)	.447** (.086)
Children (1 = have a child)	–.035 (.036)	–.042 (.036)	–.033 (.036)	.030 (.048)	.018 (.048)	.031 (.048)	–.005 (.067)	–.023 (.067)	–.002 (.067)
Age	.034^+^ (.018)	.035^+^ (.018)	.034^+^ (.018)	.058* (.025)	.059* (.025)	.058* (.025)	.092** (.034)	.093** (.035)	.092** (.034)
Age^2^	–.000 (.000)	–.000 (.000)	–.000 (.000)	–.001^+^ (.000)	–.001^+^ (.000)	–.001^+^ (.000)	–.001* (.001)	–.001* (.001)	–.001* (.001)
Gender (1 = female)	.047 (.032)	.049 (.032)	.045 (.032)	–.072^+^ (.043)	–.066 (.043)	–.072^+^ (.043)	–.029 (.060)	–.020 (.060)	–.031 (.060)
Emotional loneliness (W1)	.241** (.019)	.245** (.019)	.244** (.019)						
Social loneliness (W1)				.259** (.019)	.261** (.019)	.260** (.019)			
Overall loneliness (W1)							.306** (.019)	.311** (.019)	.309** (.019)
R^2^	.109	.108	.111	.199	.196	.199	.210	.207	.212
Observations	2,725	2,725	2,725	2,725	2,725	2,725	2,725	2,725	2,725

*Source*: Generations and Gender Survey Wave 1 (2004–2011) and Wave 2 (2007–2015).

*Note*: The coefficients are estimated via ordinary least squares. Standard errors are in parentheses. MAR = medically assisted reproduction; W1 = Wave 1; M2 = Model 2; M3 = Model 3; M4 = Model 4.

+ *p* < .1, *p < .05, **p < .01 (two-tailed tests).

### Interaction by Gender and Live Birth

**Figure 3.**
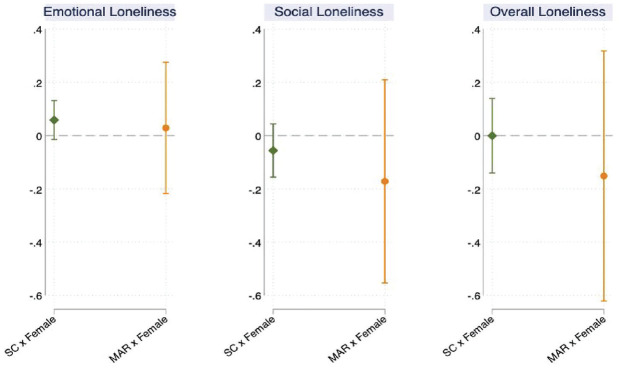
Average Marginal Effects of the Mode of Conception by Gender (with 95% Confidence Interval; *N* = 2,725). *Source:* Generations and Gender Survey Wave 1 (2004–2011) and Wave 2 (2007–2015). *Note*: SC = spontaneous conception; MAR = medically assisted reproduction.

Figure 3 presents the average marginal effects of the mode of conception on loneliness by the respondents’ gender (Hypothesis 2a and Hypothesis 2b). The full model results are reported in Appendix Table 3 in the online version of the article. The reference category for each mode of conception consists of male respondents. Regardless of the mode of conception, women experience a larger shift in emotional loneliness and a smaller change in social loneliness than their male counterparts, but the differences are not statistically different than zero. The findings support Hypothesis 2b, which suggests that the effect of undergoing MAR on changes in loneliness do not differ by gender, and not Hypothesis 2a. However, it should be noted that the confidence intervals are wide for individuals who were trying to conceive via MAR, which indicates that the differences by gender are not precisely estimated. Nonetheless, the gender differences in overall loneliness are very small, and the direction of the association for social loneliness suggests that the changes were smaller for women than for men.

**Figure 4.**
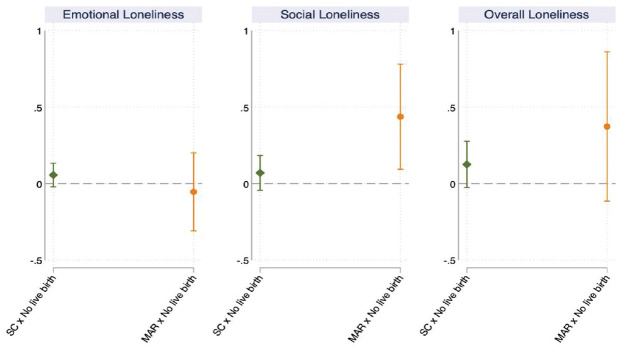
Average Marginal Effects of the Mode of Conception by Live Birth (with 95% Confidence Intervals; *N* = 2,725). *Source*: Generations and Gender Survey Wave 1 (2004–2011) and Wave 2 (2007–2015). *Note*: SC = spontaneous conception; MAR = medically assisted reproduction.

Figure 4 plots the average marginal effects of the mode of conception on loneliness depending on whether the pregnancy-seeking process did or did not end in a live birth (Hypothesis 3). The full model results are presented in Appendix Table 4 in the online version of the article. The reference category for each mode of conception consists of respondents who had at least one child between the two periods. Respondents who did not have a child after having undergone MAR experience a significantly larger increase in social loneliness than respondents who underwent MAR and had a live birth. Accordingly, among respondents who underwent MAR, not having a baby increases their social loneliness by .45 points compared to having a live birth (see Appendix Table 4 in the online version of the article), but there are no statistically significant differences in their emotional and overall loneliness depending on whether they did or did not give birth. Among respondents who were trying to conceive spontaneously, there are no statistically significant differences in both their emotional and social loneliness depending on whether they did or did not give birth. Thus, we find evidence in support of Hypothesis 3, which posits that not having a live birth as a result of MAR makes individuals to experience higher increases in loneliness.

## Discussion

Because the utilization of MAR has grown steadily in recent decades and is expected to continue to increase (Raymer et al. 2020), the mode of conception is becoming an increasingly important factor in research on reproduction. In this study, we used longitudinal data from the GGS to investigate the association between undergoing MAR and changes in loneliness among individuals in heterosexual couples. This aspect of infertility treatments has not been previously explored even though loneliness is a relevant aspect of public health and a predictor of social and mental well-being that can be experienced at various stages of the life course. Our study generated three key findings.

First, we found that the likelihood of experiencing loneliness varied by the mode of conception. Social loneliness was shown to increase more among individuals who underwent MAR to conceive than among individuals who were trying to conceive spontaneously. By contrast, we did not observe differences in emotional and overall loneliness by the mode of conception. The magnitude of the association for social loneliness was nontrivial. In additional analyses, we found that the effect of having undergone MAR on the increase in social loneliness corresponds to .036 SD, which was almost 60% of the effect of union dissolution on social loneliness (.062 SD; see Appendix Table 5 in the online version of the article).

We also investigated whether union dissolution and changes in subjective financial hardship between the two observation points mediated the association between undergoing MAR and experiencing social loneliness. The results show that while union dissolution positively and significantly predicted changes in social loneliness, it does not attenuate the association between undergoing MAR and experiencing social loneliness. Similarly, we observed that experiencing financial hardship did not mediate the increased levels of social loneliness among individuals who were undergoing MAR.

We were not able to test other potential mechanisms, including the role of mental health and stigma surrounding infertility. However, we descriptively showed that individuals who underwent MAR reported perceiving stronger social pressure to have a child from their parents, friends, and relatives than individuals who were trying to conceive spontaneously (see Figure 2) at the start of the survey. While we were not able to observe the date at which each respondent started undergoing MAR, we know that they were already receiving infertility treatment at the time of the first interview. These descriptive findings suggest that the increased levels of social loneliness observed among respondents undergoing MAR could be attributable to these individuals facing greater social pressure from their inner circle than respondents who were seeking to conceive spontaneously. This finding is consistent with the results of the existing literature, which show that individuals who undergo MAR are exposed to stigma surrounding infertility and childlessness (Greil, McQuillan, Lowry, and Shreffler 2011; Slade et al. 2007).

Second, the results did not uncover any gender differences, which may suggest that undergoing MAR treatments is a couple experience rather than an individual experience and thus has similar emotional and social consequences for both partners. An alternative explanation is that while the stigma surrounding infertility and childlessness is stronger for women than for men, women tend to talk about the problem of infertility more openly and frequently than men and receive more social support in return (Martins et al. 2014). Thus, even if women experience more loneliness than men in the initial phase of the MAR treatments, this disadvantage may be mitigated because women tend to have a stronger social support network than men. Future research should seek to shed light on how the experience of undergoing MAR varies by gender by exploring the various coping mechanisms that women and men use at different stages of the MAR treatment process.

Third, we found that the association between undergoing MAR and the levels of social loneliness individuals experienced was mainly driven by whether they did or did not have a baby between the two observation points. In other words, among couples who were trying to conceive via MAR, whether the pregnancy-seeking period did or did not result in a live birth strongly affected their feelings of loneliness. Our results confirmed the findings of Holter et al. (2021), who reported that the dominant emotional reaction among women who underwent an unsuccessful treatment attempt was the feeling of being lost and lonely.

Taken together, the findings contribute to our understanding of infertility as a socially constructed state. First, by showing that undergoing MAR is associated with an increase in social loneliness, they suggest that families and friends, rather than intimate partners, play a prominent role in shaping the loneliness aspect of the infertility experience. By this means, they underscore the importance of conceptualizing infertility—its definition, understanding, and implications—through the larger social context in which it occurs. Second, the fact that the results do not reveal differences between men and women underline that undergoing MAR is a couple experience and that both partners can be similarly affected by infertility, the lack of a desired social role, and the stigma surrounding it. Therefore, adopting a couple approach, rather than an individual one, in MAR studies can enable researchers to investigate infertility experience in a more comprehensive way. This finding further indicates that men should be an essential part of the conversation and the research on reproductive experiences and health. More broadly, the findings highlight the role of social ties and social norms in shaping treatment experience throughout the life course, one of the key underpinning of medical sociology (Umberson and Karas Montez 2010).

This study has some limitations. First, the sample of individuals who underwent MAR was small, with 198 observations. The small sample size meant that the confidence intervals were larger for MAR couples than for couples who were trying to conceive spontaneously, and it might have obscured additional differences in loneliness between the two groups. Working with a small sample also implies that we were limited in our ability to explore the relationship between MAR and loneliness by social groups and contexts. For instance, we were not able to explore the interaction between childbearing status and gender, which would otherwise have allowed us to investigate whether the feeling of loneliness associated with an unsuccessful MAR attempt differed by gender. The link between MAR and loneliness could depend on sociodemographic characteristics, such as SES, age, marital status, sexual orientation, or type of residence, because one’s reference social context shapes the social norms on reproduction (Billari et al. 2011) and the availability of social support and networks to alleviate the well-being costs of infertility (Sormunen et al. 2020).

Moreover, the relationship between MAR and loneliness can vary by country due to differences in access to infertility treatments, the generosity of the welfare state in funding treatments, and psychological support offered as part of MAR. According to the European Atlas of Fertility Policies, all countries in our sample provide partial public funding for MAR except Georgia, where no public subsidies are available for MAR patients. Psychological support within MAR process is only offered in Germany, while such service is available only for failed MAR attempts in Austria. Except Germany, the access to MAR is restricted to couples.^7^ These discrepancies might shape the MAR experience because they determine for whom and under which circumstances the treatments are accessible. In addition, the legal framework is an important aspect of the relationship between MAR and loneliness because it can influence the social norms on reproduction and MAR, and they can affect the way individuals seeks for emotional and social support. Nevertheless, even in Germany—which ranked the highest in terms of accessibility and regulation of MAR among the countries in our sample^8^—high-income couples who experience subfertility are more likely to seek medical help to have a child than their lower-income counterparts (Köppen et al. 2021). To the extent that the profiles of individuals who undergo MAR are similar across countries, the association between undergoing MAR and experiencing loneliness may not vary substantially across contexts.

Second, we were not able to observe the loneliness levels of the respondents before the pregnancy-seeking period. Hence, the association we found between undergoing MAR and experiencing loneliness was potentially a conservative estimate given that individuals who underwent MAR to conceive might have been dealing with infertility issues for a longer period of time than couples who were trying to conceive spontaneously, and this difference might have been reflected in their baseline loneliness levels. In other words, respondents who were trying to conceive before the survey period might have already experienced a substantial increase in loneliness. Moreover, we do not know when the MAR treatment started, how long it lasted, and whether there had been any interruptions or failed attempts. All these aspects of fertility treatments could influence the baseline loneliness level that is measured in Wave 1. When we look at the baseline loneliness levels reported in Table 1, we can see that individuals who underwent MAR experienced higher levels of emotional and overall loneliness than individuals who conceived spontaneously, although the mean difference was not statistically significant. Also, we were not able to identify individuals who suffered from infertility but who did not access MAR (for a review of the literature on who is more likely to be in this group, see Passet-Wittig and Greil 2021), which also potentially resulted in our estimates being conservative.

Third, we restricted our analytical sample to respondents who indicated that they “want to have a/another baby” at the moment of the interview in Wave 1. Conceptually, wanting to have a baby may correlate but not necessarily fully overlap with seeking to become pregnant. Therefore, we might have selected respondents who were not actively seeking to achieve a pregnancy, which might have introduced biases into our analysis because they are likely to be more frequent among respondents who were trying to conceive spontaneously.

These limitations are offset by several strengths of our study. Our study broadens the perspective of MAR research by covering for the first time the aspect of loneliness and by showing that seeking to conceive through MAR is associated with an increase in social loneliness among heterosexual couples in Europe. This is an important finding because loneliness is a significant aspect of population health and is an important indicator of social well-being that can exacerbate the risk of morbidity and mortality. Moreover, by relying on a rich longitudinal data set, we were able to show that the association was mainly driven by individuals who did not give a live birth and that the effects were not more pronounced for women than for men. Finally, accounting for potential confounding factors strengthened the robustness of our findings.

## Supplemental Material

sj-docx-1-hsb-10.1177_00221465231167847 – Supplemental material for Loneliness during the Pregnancy-Seeking Process: Exploring the Role of Medically Assisted Reproduction Click here for additional data file. Supplemental material, sj-docx-1-hsb-10.1177_00221465231167847 for Loneliness during the Pregnancy-Seeking Process: Exploring the Role of Medically Assisted Reproduction by Selin Köksal and Alice Goisis in Journal of Health and Social Behavior

## Notes

1. The International Glossary on Infertility and Fertility Care defines MAR as “reproduction brought through various interventions, procedures, surgeries and technologies to treat different forms of fertility impairment and infertility” (Zegers-Hochschild et al. 2017:1796).

2. These countries include Bulgaria, Georgia, Germany, Austria, and Poland.

3. Note that the participant countries were interviewed in different years but within the indicated time interval.

4. We excluded Lithuania from our sample due to high levels of attrition (≈52%).

5. We selected respondents with a coresident or a nonresident partner because the question on the mode of conception was only posed to partnered participants. This led to a minor gender imbalance (52.5% women vs. 47.5% men) in the analytical sample because women are more likely to be partnered.

6. Breakdown by the type of treatment: receiving medication (66.9%), IVF (6.1%), surgery (1.5%), artificial insemination (2.6%), other medical treatment (6.1%), uses method but type is not specified (16.9%).

7. Legally, MAR treatments are only accessible for heterosexual couples in Poland and Georgia, whereas homosexual female couples are eligible for MAR in Germany, Austria, and Bulgaria.

8. For detailed information on fertility treatment policies in Europe, see the European Atlas of Fertility Treatment Policies at https://fertilityeurope.eu/european-atlas-of-fertility-treatment-policies/.
