# Supplementary material for: Loneliness during the Pregnancy-Seeking Process: Exploring the Role of Medically Assisted Reproduction
Source: J Health Soc Behav. 2023 May 5;64(2):209–27. doi: 10.1177/00221465231167847 (PMC10230592; doi:10.1177/00221465231167847)
Supplement: sj-docx-1-hsb-10.1177_00221465231167847 – Supplemental material for Loneliness during the Pregnancy-Seeking Process: Exploring the Role of Medically Assisted Reproduction [file sj-docx-1-hsb-10.1177_00221465231167847.docx]

**Journal** of **Health**

and **Social Behavior**

OFFICIAL JOURNAL OF THE AMERICAN SOCIOLOGICAL ASSOCIATION

**ONLINE SUPPLEMENT**

**to article in**

Journal of Health and Social Behavior

**Loneliness during the Pregnancy-Seeking Process: Exploring the Role of Medically Assisted Reproduction**

**Selin Köksal**

*Institute for Social and Economic Research, University of Essex*

**Alice Goisis**

*Centre for Longitudinal Studies, University College London*

**Appendix Table 1:** Mode of conception by gender

|  | **Female** | **Male** |
| --- | --- | --- |
|  | *(%)* | |
| ***Mode of conception*** |  |  |
| Spontaneous conception | 92.5 | 87.8 |
| MAR conception | 4.3 | 9.5 |
| Missing | 3.2 | 2.7 |
| ***Total*** | 100 | 100 |

**Appendix Table 2:** Mean level of education by mode of conception and live birth

|  | **SC x Live birth** | **MAR x Live birth** | **SC x No live birth** | **MAR x No live birth** |
| --- | --- | --- | --- | --- |
|  |  |  |  |  |
| Secondary or below | 0.67 | 0.60 | 0.66 | 0.66 |
| Higher | 0.33 | 0.40 | 0.34 | 0.34 |
|  |  |  |  |  |
| *N. obs* | 940 | 78 | 1570 | 118 |

*Note*: SC = Spontaneous Conception; MAR = Medically Assisted Reproduction

**Appendix Table 3:** Average marginal change in loneliness by mode of conception and gender

|  | (1) | (2) | (3) |
| --- | --- | --- | --- |
| VARIABLES | Emotional | Social | Overall |
|  |  |  |  |
| SC x Female *(ref: SC x Male)* | 0.053 | -0.057 | -0.007 |
|  | (0.033) | (0.044) | (0.062) |
| MAR x Female *(ref: MAR x SC Male)* | 0.021 | -0.188 | -0.177 |
|  | (0.125) | (0.168) | (0.234) |
| Controls | Yes | Yes | Yes |
| Observations | 2,725 | 2,725 | 2,725 |
| Mean difference (SC) | 0.02 | 0.04 | 0.05 |
|  |  |  |  |
| Mean difference (MAR) | -0.01 | 0.23 | 0.22 |
|  |  |  |  |

Note: Standard errors in parentheses. ** p<0.01, * p<0.05, + p<0.1 SC = Spontaneous Conception; MAR = Medically Assisted Reproduction

**Appendix Table 4:** Average marginal change in loneliness by mode of conception and live birth

|  | (1) | (2) | (3) |
| --- | --- | --- | --- |
| VARIABLES | Emotional | Social | Overall |
|  |  |  |  |
| SC x No live birth *(ref: SC x Live birth)* | 0.053 | 0.065 | 0.117+ |
|  | (0.036) | (0.049) | (0.068) |
| MAR x No live birth *(ref: MAR x Live birth)* | -0.036 | 0.451** | 0.405+ |
|  | (0.120) | (0.161) | (0.224) |
| Controls | Yes | Yes | Yes |
| Observations | 2,725 | 2,725 | 2,725 |
| Mean difference (SC) | 0.02 | 0.04 | 0.05 |
| Mean difference (MAR) | -0.01 | 0.23 | 0.22 |

Note: Standard errors in parentheses. ** p<0.01, * p<0.05, + p<0.1 SC = Spontaneous Conception; MAR = Medically Assisted Reproduction

**Appendix Table 5:** The association between union dissolution and loneliness and the association between the mode of conception and loneliness. Standardized beta coefficients.

|  | (1) | (2) | (3) |
| --- | --- | --- | --- |
| VARIABLES | Emotional | Social | Total |
| *Panel A* |  |  |  |
| Union dissolution | 0.067 | 0.062*** | 0.077 |
| *Panel B* |  |  |  |
| MAR | -0.011 | 0.036* | 0.020 |
| Baseline loneliness | Yes | Yes | Yes |
| Socio-demographics | Yes | Yes | Yes |
| Country fixed effects | Yes | Yes | Yes |
|  |  |  |  |
| Observations | 2,669 | 2,669 | 2,669 |
|  |  |  |  |
